# Effect of a Polysaccharide from Cultivated *Buchwaldoboletus* sp. on DSS-Induced Ulcerative Colitis in Mice

**DOI:** 10.3390/nu18142225

**Published:** 2026-07-08

**Authors:** Liyuan Ba, Jingyi Tu, Yirong Mao, Yihui Li, Liping Sun

**Affiliations:** Faculty of Food Science and Engineering, Kunming University of Science and Technology, Kunming 650500, China; 18313104941@163.com (L.B.); 17380573809@163.com (J.T.); maoyirong0002@163.com (Y.M.); m14736806038@163.com (Y.L.)

**Keywords:** mushroom, polysaccharidces, ulcerative colitis, inflammatory, gut microbiota

## Abstract

Background: In this study, a cultivated mushroom (*Buchwaldoboletus* sp.) polysaccharide (BSP60) was prepared. Methods: The protective effect of BSP60 on ulcerative colitis (UC) was evaluated using a dextran sulphate sodium (DSS)-induced murine model of UC. Results: The results showed that BSP60 significantly alleviated colonic inflammation and oxidant stress in DSS-treated mice. BSP60 reduced the overproduction of TNF-α, IL-1β, and IL-6; lowered the accumulation of MDA and LPS in serum and colon; and increased the activities of antioxidant enzymes SOD and GSH-Px in colonic tissues. Histological observations revealed that BSP60 significantly alleviated DSS-induced colonic tissue damage in mice by reducing inflammatory cell infiltration and mucosal injury. Immunofluorescence staining revealed that BSP60 increased the expression of intestinal barrier-associated proteins ZO-1, occludin, and claudin-1 in DSS-induced UC mice, thereby alleviating inflammation. Analysis of 16S rRNA gene sequencing indicated that BSP60 maintained gut microbiota homeostasis in UC mice and inhibited the excessive growth of inflammation-associated microorganisms. BSP60 also enhanced the production of short-chain fatty acids in the mice feces. The protective effect of BSP60 on UC was dose dependent. Conclusions: BSP60 exerts anti-UC effects, providing a scientific basis for its application as a functional food ingredient to improve intestinal health.

## 1. Introduction

Ulcerative colitis (UC) is an inflammatory bowel disease (IBD) characterized by superficial ulcers in the rectum and distal colon [1]. Its pathogenesis is multifactorial, with the gut microbiota playing a key role [2]. In UC, harmful bacteria and their toxins penetrate the damaged mucosal barrier, reaching intestinal epithelial cells and underlying immune cells. The immune system recognizes bacterial components via pattern recognition receptors, inducing the massive release of pro-inflammatory cytokines by immune cells [3]. These cytokines recruit neutrophils and macrophages into the intestinal mucosa [4]. While eliminating pathogens, these cells release reactive oxygen species (ROS) and proteolytic enzymes, causing severe oxidative damage to epithelial cells [3,5]. This phenomenon leads to the extensive death of epithelial cells, downregulation of tight junction proteins (e.g., ZO-1, occludin, and claudin-1), and formation of superficial ulcers. If dysbiosis persists, then a vicious cycle ensues: A compromised barrier permits ongoing bacterial invasion, sustaining immune activation and chronic inflammation.

In addition to utilizing drugs such as aminosalicylates, corticosteroids, 6-mercaptopurine, and specific biologics to inhibit the immune response, the treatment of IBD now focuses on modulating gut microbiota. Some studies demonstrated that the long-term administration of immunosuppressive drugs may induce various complications, including antibiotic resistance, osteoporosis, and anemia, and exacerbate gastrointestinal ulcers and other symptoms [6,7,8]. Exploring natural and safe substances for UC intervention and adjustment is necessary. Certain natural substances, such as peptides [3,9], polyphenols [10], probiotics [11], and polysaccharides [12], can effectively alleviate and intervene in UC.

Polysaccharides possess excellent biocompatibility and safety, exhibit significant antioxidant and anti-inflammatory properties, and have a prebiotic effect on beneficial gut microbiota; therefore, they demonstrate significant therapeutic potential for UC. Li et al. [13] demonstrated that *Hericium erinaceus* fruiting body polysaccharide (HEFP) reduced oxidative stress in UC mice, regulated pro-inflammatory cytokines, and recovered the gut microbiota. Wei et al. [14] isolated and purified a neutral polysaccharide named BAP1-1 from *Boletus aereus*; BAP1-1 improved microbial ecological imbalance and obviously protected the damaged mucus barrier in colitis mice. Lu et al. [15] compared the alleviating effects and underlying mechanisms of polysaccharides from *Cyclocarya paliurus* (Batal.) Iljinskaja (CP) and Chinese yam (*Dioscorea opposita* Thunb.) polysaccharide (CYP) on DSS-induced UC in mice. Wu et al. [16] showed that the morel stems crude polysaccharides regulated colonic damage, decreased inflammation, and promoted tissue repair and short-chain fatty acid (SCFA) production in the intestines of UC mice.

*Buchwaldoboletus* sp. (BS) is a cultivated edible mushroom. Our previous research analyzed the basic nutritional properties of BS and extracted fruiting body polysaccharides BSP60, BSP40, and BSPW using a water-extraction, ethanol-solubilization method [17]. We found that BSP60 possesses extremely high antioxidant activity and potent in vitro anti-inflammatory activity and exhibits a high prebiotic effect. To further explore the physiological activity of BSP60, this study investigated the potential of BSP60 in the intervention and alleviation of UC in vivo and determined its underlying mechanisms of action.

## 2. Materials and Methods

### 2.1. Materials and Reagents

BS was procured from the Yunnan Mushroom Trading Centre (Kunming, China), and the polysaccharide component BSP60 was extracted from the fruiting bodies of BS using the methods described in our previous study [17]. Dextran sulphate sodium (DSS) was provided by MP Biomedicals (Santa Ana, CA, USA). Mesalazine was supplied by Sigma (St. Louis, MO, USA). Interleukin (IL)-1β, IL-6, IL-10, TNF-α, Lipopolysaccharide (LPS), Superoxide Dismutase (SOD), Malondialdehyde (MDA), and Glutathione peroxidase (GSH-Px) ELISA kits were acquired from Shanghai Duma Co., Ltd. (Shanghai, China). ZO-1, occluding, and claudin-1 antibodies were obtained from Jinghe Biotechnology Co., Ltd. (Guangdong, China).

### 2.2. Experimental Design

Fifty male SPF-grade C57BL/6J mice (7 weeks old, 20 ± 2 g) were provided by Beijing Vitonlihu Laboratory Animal Technology Co., Ltd. (Beijing, China) housed in a room maintained at 22 ± 2 °C with a 12-h light–dark cycle, given free access to food and water, and acclimatized for 1 week. Each cage of mice was considered as one experimental unit.

All animal experiments were conducted according to the relevant guidelines and regulations and were approved by the Committee on Animal Welfare and Ethics at Kunming University of Science and Technology (Kunming, China), with Approval No.: SLWH-KUST-20250096. After a 1-week acclimatization period, the mice were randomly divided into five groups: normal control (NC), model (MC), positive control (PC), low-dose BSP60 (L-BSP60), and high-dose BSP60 (H-BSP60). Group allocation was performed by an independent researcher using a random number table and sealed envelopes, with the operators kept blinded to group assignments. All animal care and experimental staff remained blinded throughout the experiment. Outcome assessments were conducted by trained researchers who were unaware of the group allocation, and data analysis was performed jointly by the authors of this manuscript. The size of *n* = 10 per group was determined according to the related studies using the same mice model [18,19]. Then the NC group was administered saline by gavage and given normal drinking water from days 1 to 14. The MC group was administered saline by gavage and given normal drinking water from days 1 to 7, then administered saline by gavage and given 2.5% DSS in drinking water from days 8 to 14. The PC group was administered mesalazine (200 mg/kg/day) by gavage and given normal drinking water from days 1 to 7, then administered mesalazine (200 mg/kg/day) by gavage and given 2.5% DSS in drinking water from days 7 to 14 [20]. The L-BSP60 group was administered BSP60 (200 mg/kg/day) by gavage and given normal drinking water from days 1 to 7, then administered BSP60 (200 mg/kg/day) by gavage and given 2.5% DSS in drinking water from days 7 to 14. The H-BSP60 group was administered BSP60 (400 mg/kg/day) by gavage and given normal drinking water from days 1 to 7, then administered BSP60 (400 mg/kg/day) by gavage and given 2.5% DSS in drinking water from days 7 to 14. On days 13–14 of the experiment, feces were collected fresh from each individual mouse separately and then immediately flash-frozen in liquid nitrogen and subsequently transferred to and stored at −80 °C until subsequent analysis. The elapsed time between defecation and freezing was strictly controlled to less than 10 min. On the evening of day 14, the mice were fasted for 12 h. Next, all mice were euthanized under isoflurane anesthesia via cardiac puncture, followed by cervical dislocation as a secondary method to ensure irreversible death. Euthanasia was confirmed by (1) cessation of heartbeat, (2) respiratory arrest, and (3) absence of response to toe pinch. After the sacrifice, the liver, kidneys, spleen, and thymus were immediately collected, flash-frozen in liquid nitrogen, and subsequently stored at −80 °C.

### 2.3. Measurement of Disease Activity Index (DAI)

The DAI of mice was scored according to the method described by Qin et al. [21], and the specific requirements are outlined in Table 1. Quantitative assessments were performed on fecal consistency (0–4 points), weight loss (0–4 points), and fecal bleeding (0–4 points). The mice with a DAI score of ≥6 points were considered to be successfully established models.

### 2.4. Measurement of Organ Indices

The surface bloodstains of the liver, kidneys, and thymus were first rinsed off with saline. The organs were then blotted dry with filter paper and weighed. The organ index of the mice was calculated using Formula (1):


(1) $$\begin{array}{c} Organ\;index\;(\%)=\;\frac{\;\mathrm{wet}\;\mathrm{weight}\;\mathrm{of}\;\mathrm{the}\;\mathrm{corresponding}\;\mathrm{organ}}{\mathrm{mouse}\;\mathrm{body}\;\mathrm{weight}}\times 100. \end{array}$$

### 2.5. Detection of Inflammatory Cytokines and Lipopolysaccharide (LPS)

Blood was collected and centrifuged at 1000× *g* for 10 min, and the serum was obtained. The concentrations of pro-inflammatory cytokines (IL-1β, TNF-α, and IL-6) and LPS in the serum were determined in accordance with the manufacturer’s instructions for the corresponding kits.

In brief, 0.1 g of colon tissue was accurately weighed, and 0.9% saline was added at a ratio of 1:9 (*w*/*v*) ratio. The mixture was homogenized in an ice-water bath, followed by centrifugation at 4 °C and 9000× *g* for 10 min. The supernatant was collected, and the levels of IL-1β, TNF-α, IL-6, and LPS were determined following the manufacturer’s instructions for the corresponding kits.

### 2.6. Determination of Oxidative Stress Markers in Serum and Colon Tissue

The activities of SOD and GSH-PX and the contents of MDA in mouse serum and colon were determined according to the manufacturer’s instructions for the corresponding kits.

### 2.7. Histopathological Analysis of Colon Tissue

Hematoxylin and eosin (H&E) and Alcian blue-periodic acid–Schiff (AB-PAS) staining were analyzed in accordance with the method reported by Zhong et al. [22] Mouse colons were fixed in 4% paraformaldehyde solution. After fixation, the tissues were dehydrated in ethanol, cleared in xylene, embedded in paraffin, cut into 5 μm-thick sections, and stained with H&E and AB-PAS. The morphological characteristics of the colon tissues were observed, and images were determined using a BX-53 microscope (Olympus Corporation, Tokyo, Japan).

### 2.8. Analysis of ZO-1, Occludin, and Claudin-1 Expression Levels in Colon Tissues

Immunofluorescence staining was used to determine the expression of ZO-1, occludin, and claudin-1 in colon tissues [23]. Paraffin sections were deparaffinized, rehydrated, and subjected to antigen retrieval in Ethylene diamine tetraacetic acid buffer (pH 8.0). The sections were washed with Phosphate buffered saline, blocked by 5% Bovine serum albumin at room temperature for 30 min, and incubated with primary antibodies (ZO-1, occludin, and claudin; 1:200) overnight at 4 °C. Next, the sections were incubated with CY3-conjugated secondary antibody (1:500) for 10 min at room temperature in the dark, followed by 4′,6-diamidino-2-phenylindole staining for 10 min. After mounting, the slides were visualized under a fluorescence microscope (CX33, Olympus Corporation, Shinjuku-ku, Tokyo, Japan).

### 2.9. Gut Microbiota Analysis

Gut microbial composition was analyzed though 16S rRNA gene amplicon sequencing [23]. Sequences were determined by Shanghai Haoweitai Biotechnology Co., Ltd. (Shanghai, China) and analyzed by 2 × 250 bp paired-end sequencing using an Illumina NovaSeq 6000 platform (Illumina (China) Scientific Co., Ltd., Shanghai, China). The resulting data were used for subsequent bioinformatics analysis.

### 2.10. Determination of Short-Chain Fatty Acids (SCFAs)

The SCFAs determined according to previous study [23]. In brief, 0.2 g of fecal samples was accurately weighed, mixed with 1 mL of ultrapure water, vortexed for 2 min, and centrifuged at 9000× *g* for 10 min. Then, 0.6 mL aliquot of the supernatant was mixed sequentially with 0.2 mL of 50% H_2_SO_4_ and 0.8 mL of ethyl acetate, vigorously shaken to mix thoroughly, incubated at −40 °C for 1 h, and centrifuged again (9000× *g*, 4 °C, 10 min). The supernatant was collected for analysis.

SCFAs were analyzed on a Shimadzu GC-MS QP-2010 system (Shimadzu Corporation, Kyoto, Japan) with an HP-FFAP column (30 m × 0.32 mm, 0.25 μm). The GC conditions were as follows: injector temperature, 240 °C; column temperature program, initial temperature 100 °C, increased to 140 °C at 6 °C/min and then to 200 °C at 15 °C/min with a 3 min hold; carrier gas, He (1.0 mL/min); injection volume, 1 μL; and split ratio, 1:20. The MS conditions were as follows: electron impact (EI) ionization, 70 eV; and ion source temperature, 200 °C. The SCFAs were quantitatively analyzed by comparing their retention times and mass fragmentation patterns with those of reference standards and by matching the acquired mass spectra against the NIST 14 database. Quantitative determinations were conducted using the external standard method, in which a standard curve was established via gradient concentrations of reference standards to quantify each component in the samples.

### 2.11. Statistical Analysis

Results were expressed by mean ± standard deviation (SD). Statistical analyses were performed using SPSS version 20.0. Comparisons among multiple groups were conducted using one-way analysis of variance (ANOVA), followed by Tukey’s HSD post-hoc test for pairwise comparisons. A *p*-value < 0.05 was considered statistically significant.

## 3. Results and Discussion

### 3.1. Effects of BSP60 on Basic Symptoms in DSS-Induced UC Mice

**Figure 1:**
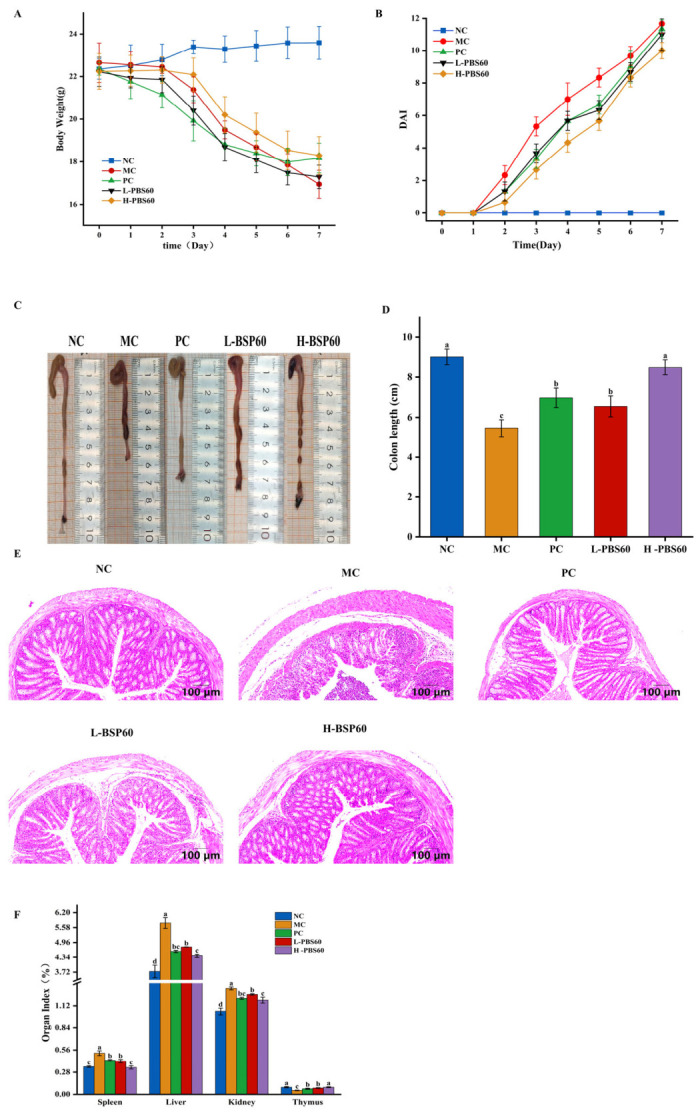
Effects of BSP60 on physiological and pathological phenotypes in DSS-induced UC mice. (**A**): Body weight changes; (**B**): Disease activity index (DAI); (**C**): Gross appearance of mouse colon; (**D**): Mouse colon length; (**E**): H&E staining of mouse colon; (**F**): Organ indices. The same letters indicate no significant difference (*p* > 0.05) and different letters indicate significant difference (*p* < 0.05).

As shown in Figure 1A,B, the mice administered with 2.5% DSS for 7 days exhibited significant body weight loss and a continuous increase in DAI compared with the NC group (*p* < 0.05). On day 4, the mice DAI score in the MC group reached 7, confirming the successful establishment of DSS-induced UC model. The mice in the PC, L-BSP60, and H-BSP60 groups showed a tendency of weight loss, but the magnitude of weight loss in these three groups was lower than that in the MC group. On days 1–2, the H-BSP60 group exhibited a significant intervention effect. Compared with that in the MC group, the increasing trend of DAI scores in the PC, L-BSP60, and H-BSP60 groups was attenuated. On day 4 of DSS induction, the DAI scores of the three intervention groups were 5.67, 5.67, and 4.33.

Colorectal shortening can reflect the extent of colon damage in DSS-induced colitis [24]. The colon length in each group is shown in Figure 1C,D. Compared with that in the NC group (9.03 cm), the average colon length in the MC group was 5.45 cm, representing significant shortening (*p* < 0.05). This finding indicates that DSS induces severe inflammation and structural damage to mouse colon. After intervention with mesalazine and BSP60, the DSS-induced structural damage was markedly attenuated, with colon lengths of 6.98, 6.55, and 8.50 cm in the PC, L-BSP60, and H-BSP60 groups, respectively. The effect of H-BSP60 was significantly higher than that of L-BSP60. A finding similar was reported by Wu et al. [16], who showed that morel polysaccharides restored the colon length in mice. This result indicates that BSP60 effectively protects colonic mucosal integrity and alleviates pathological damage in mice receiving oral DSS.

Figure 1E shows the H&E staining of colonic tissues from each group. The mice in NC group exhibited intact colonic structure with continuous mucosal epithelium and neatly arranged crypts. The MC group showed severe colonic damage, characterized by extensive inflammatory cell infiltration in the mucosal layer, epithelial disruption, and disorganized or even widespread crypt loss. After intervention with PC, L-BSP60, and H-BSP60, these pathological changes were alleviated to varying degrees. BSP60 improved colonic tissue damage in a dose-dependent manner. The H-BSP60 group showed the highest improvement with an intact colonic structure, a smooth mucosal epithelium, tightly arranged crypts, and no evident inflammatory cell infiltration; its morphology was close to that of the NC group. Although the L-BSP60 group still exhibited a small amount of inflammatory cell infiltration and a slight reduction in crypts, the colonic structure remained largely intact. The protective effect of BSP60 against colonic tissue damage is consistent with the findings of Wei et al. [14], who reported that boletus polysaccharide (BAP1-1) could alleviate DSS-induced colonic injury.

As shown in Figure 1F, the spleen, thymus, liver, and kidney indices were further measured. Compared with the NC group, the MC group showed significantly increased spleen, liver, and kidney indices (*p* < 0.05) but significantly decreased thymus index (*p* < 0.05). After intervention with BSP60, these DSS-induced abnormalities were alleviated in a dose-dependent manner. In particular, the spleen, liver, and kidney indices in the L-BSP60 group were lower than those in the MC group, whereas the thymus index increased. In the H-BSP60 group, all indices differed significantly from those in the MC group (*p* < 0.05) and showed no significant difference from those in the NC group. The protective effect of BSP60 on spleen index is similar to the report by Liu et al. [25], who found that *Platycodon* polysaccharides significantly reduced spleen index in a mouse model of UC.

### 3.2. Effects of BSP60 on Inflammatory Cytokines of the Serum and Colon in DSS-Induced UC Mice

**Figure 2:**
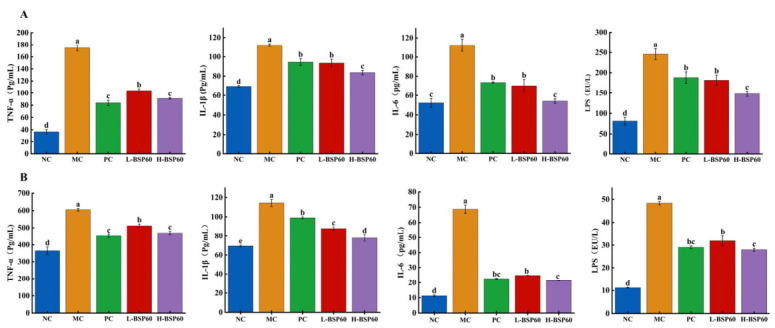
Levels of inflammatory cytokines and endotoxin (LPS) in serum (**A**) and colon homogenates (**B**) of mice. The same letters indicate no significant difference (*p* > 0.05) and different letters indicate significant difference (*p* < 0.05).

Figure 2A show the serum levels of inflammatory cytokines and endotoxin (LPS) in each group. The excessive release of pro-inflammatory cytokines and the resulting inflammatory cascade are key events in UC pathogenesis [26]. Compared with the NC group, the MC group showed significantly higher levels of TNF-α, IL-1β, IL-6, and LPS with values of 174.78 pg/mL, 112.15 pg/mL, 112.45 pg/mL, and 245.78 EU/L, respectively (*p* < 0.05). H-BSP60 lowered IL-1β to 83.67 pg/mL, IL-6 to 54.12 pg/mL, and LPS to 147.86 EU/L; all these values were lower than those in the PC and MC groups. In particular, the IL-6 level was almost similar to that in the NC group. The TNF-α level in the H-BSP60 group was 91.07 pg/mL, slightly higher than that in the PC group but still significantly below that in the MC group (*p* < 0.05), suggesting a selective regulation effect. These findings are consistent with the report of Wu et al. [16], who found that crude polysaccharides from morel mushroom stems reduced serum TNF-α and IL-6 levels in mice with DSS-induced colitis.

Figure 2B show the levels of inflammatory cytokines and endotoxin (LPS) in colonic tissue homogenates from each group. Compared with the NC group, the MC group had significantly higher levels of TNF-α, IL-1β, IL-6, and LPS in colon tissues with values of 606.93 pg/mL, 114.34 pg/mL, 68.69 pg/mL, and 48.36 EU/L, respectively. After BSP60 intervention, all inflammatory markers dose-dependently decreased. The TNF-α level in the H-BSP60 group was not significantly different from that in the PC group and was significantly lower than that in the MC group (*p* < 0.05). The IL-1β level in the H-BSP60 group was 77.95 pg/mL, significantly lower than those in the PC and MC groups (*p* < 0.05). The IL-6 level in the L-BSP60 group was not significantly different from that in the PC group (*p* > 0.05). L-BSP60 had a similar effect on LPS and IL-6. Lu et al. [15] showed that *Cyclocarya paliurus* (Batal.) Iljinskaja (CP) and Chinese yam (Dioscorea opposita Thunb.) polysaccharide (CYP) increased colitis symptoms in DSS-induced mice and inhibited cytokines (IL-1β, TNF-α). Li et al. showed that HEFP reduced colonic IL-1β and IL-6 levels. These findings are similar to our results.

### 3.3. Effects of BSP60 Intervention on Oxidative Stress in DSS-Induced UC Mice

**Figure 3:**
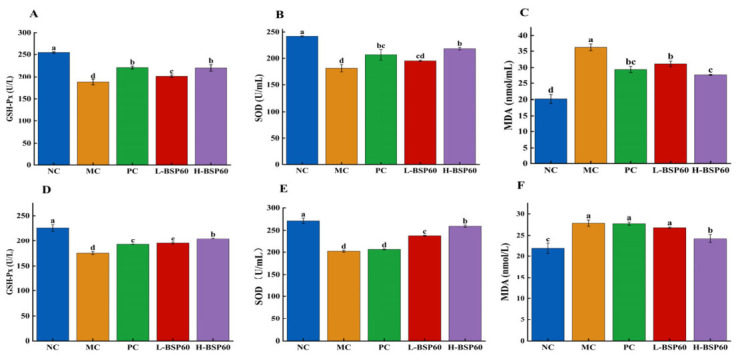
Levels of oxidative stress markers in mouse serum (**A**) GSH-Px, (**B**) SOD, (**C**) MDA and colon homogenates (**D**) GSH-Px, (**E**) SOD, (**F**) MDA. The same letters indicate no significant difference (*p* > 0.05) and different letters indicate significant difference (*p* < 0.05).

Regulating oxidative stress is a key mechanism in alleviating UC. Imbalances in antioxidant enzyme (GSH-Px and SOD) activities and lipid peroxidation product (MDA) levels in serum and colon tissues are markers of colon tissue damage and leukocyte infiltration [27]. Figure 3 shows oxidative stress markers in serum and colon tissues.

The serum oxidative stress markers are shown in Figure 3A–C. Compared with the NC group, the MC group exhibited significantly lower GSH-Px activity (from 254.38 U/mL in the NC group to 187.45 U/mL) and SOD activity (from 242.11 U/mL in the NC group to 181.85 U/mL) but significantly increased MDA content (from 20.16 nmol/mL in the NC group to 36.18 nmol/mL) (*p* < 0.05). This finding indicates that DSS successfully induces systemic oxidative stress. Compared with the MC group, the L-BSP60 group had significantly increased GSH-Px activity and SOD activity and decreased MDA content (*p* < 0.05). Compared with the L-BSP60 group, the H-BSP60 group exhibited significantly increased GSH-Px and SOD activities and decreased MDA content (*p* < 0.05). Therefore, the oxidative stress markers were dose-dependently improved.

The oxidative stress markers in colon showed trends largely consistent with those in serum. Compared with that of the NC group, the colon tissues of the MC group had significantly decreased GSH-Px activity (from 226.41 U/mL in the NC group to 175.22 U/mL, *p* < 0.05) and SOD activity (from 271.33 U/mL in the NC group to 202.01 U/mL, *p* < 0.05) but significantly increased MDA content (from 21.84 nmol/mL in the NC group to 27.87 nmol/mL, *p* < 0.05). After BSP60 intervention, the GSH-Px activity in the L-BSP60 group increased to 195.32 U/mL (*p* < 0.05), the SOD activity increased to 238.06 U/mL (*p* < 0.05), and the MDA content decreased to 26.81 nmol/mL (*p* < 0.05). The effect of H-BSP60 was significantly higher than that of L-BSP60. These beneficial effects of BSP60 on colonic oxidative stress markers are consistent with the report by Ma et al. [28], who demonstrated that acetylated morel polysaccharide increased SOD, CAT, and GSH-Px activities and reduced MDA levels in colonic tissues. Li et al. [13] studied that HEFPs reduced oxidative stress in UC mice by decreasing MDA levels and increasing SOD and catalase activities. These findings are in accordance with our results.

### 3.4. Effects of BSP60 on Colonic Tissue Pathological Damage in DSS-Induced UC Mice

**Figure 4:**
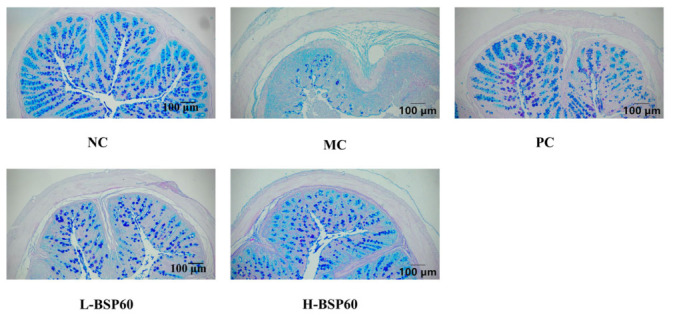
AB-PAS staining of mouse colon tissue in each group.

AB-PAS staining can visualize the mucins present in the mucus layer of colonic epithelial cells and mucin glycoproteins stored in the secretory granules of goblet cells [29]. Figure 4 shows that within the same field of view, the number of goblet cells in colon was significantly lower in MC mice than in NC mice, and the mucus layer appeared thinned or even disrupted, indicating that oral DSS severely impairs the integrity of the colonic mucus barrier. After treatment with mesalazine and BSP60, the reduction in goblet cell numbers was attenuated to varying degrees in the PC, L-BSP60, and H-BSP60 groups. The stained mucus layer became darker, and its continuity improved, with the most pronounced effect observed in the H-BSP60 group. These reparative effects of BSP60 on goblet cells and the mucus barrier are consistent with the report by Zou et al. [30], who demonstrated that Huangshan flower mushroom polysaccharides (FMPS) increased colonic goblet cell numbers in UC mice. Therefore, BSP60 effectively promotes goblet cell proliferation and mucus secretion, thereby repairing mucus barrier damage.

**Figure 5:**
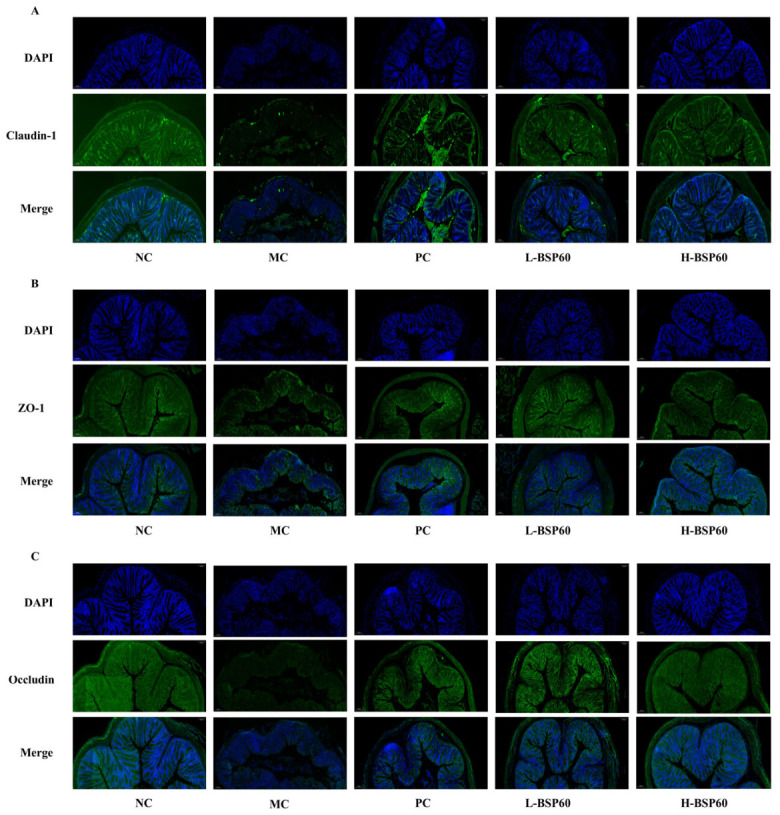
Effect of BSP60 on the expression of tight junction proteins in the colon tissue of DSS-induced UC mice. (**A**): Claudin-1; (**B**): ZO-1; (**C**): Occludin.

Figure 5 shows the effect of BSP60 on tight junction (TJ) proteins in the colon of DSS-induced colitis mice. Epithelial TJ proteins, such as closed small band-1 protein (ZO-1) and transmembrane proteins (claudin-1 and occludin), maintains intestinal mucosal barrier function and regulate permeability to water, ions, and nutrients [31]. Immunofluorescence staining showed that claudin-1, ZO-1, and occludin formed a continuous linear pattern along the cell membrane with high intensity in the NC group, indicating intact TJs. In the MC group, the fluorescence intensity was markedly reduced, and the linear pattern was disrupted into a diffuse, punctate distribution, indicating severe TJ damage. After mesalazine or BSP60 treatment, the fluorescence intensity and membrane continuity were significantly restored. BSP60 could upregulate claudin-1, ZO-1, and occludin, promote their correct membrane localization, and effectively repair the intestinal epithelial barrier in UC mice. Wu et al. [16] reported that morel polysaccharides upregulated the TJ proteins in UC mice. Lu et al. [15] studied the integrity of intestine by improving the expression of mucin ZO-1 and occludin. These reports are consistent with our results.

These results demonstrate that BSP60 effectively repairs intestinal barrier damage in DSS-induced colitis mice, as evidenced by the restored goblet cell numbers and mucus secretion (Figure 4) and upregulated expression and proper localization of TJ proteins claudin-1, ZO-1, and occludin (Figure 5). By synergistically repairing the mucus and tight junction barriers, BSP60 maintains intestinal epithelial integrity, providing a structural basis for its protective effects against UC. The effect of BSP60 is dose dependent.

### 3.5. Effects of BSP60 on the Gut Microbiota of DSS-Induced Colitis Mice

**Figure 6:**
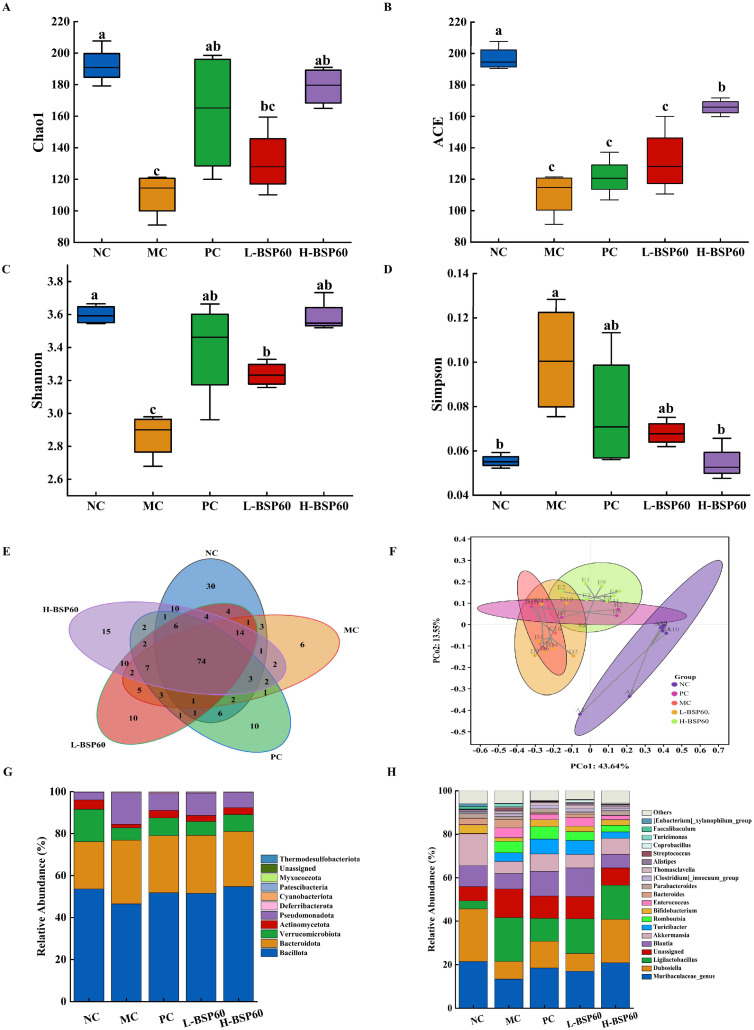
Effects of BSP60 on gut microbiota in DSS-induced UC mice. (**A**): Chao1 index; (**B**): ACE index; (**C**): Shannon index; (**D**): Simpson’s index; (**E**): Venn diagram; (**F**): Principal coordinate analysis (PCoA); (**G**): Relative abundance at the phylum level; (**H**): Relative abundance at the genus level. The same letters indicate no significant difference (*p* > 0.05) and different letters indicate significant difference (*p* < 0.05).

The gut microbiota plays an important role in metabolizing nondigestible polysaccharides by exerting metabolic, immunological, and protective functions [32]. In this study, 16S rRNA gene sequencing was performed to determine whether the amelioration of DSS-induced colitis is associated with changes in gut microbial structure. As shown in Figure 6A–D, the ACE and Chao1 indices of the gut microbiota in MC mice were significantly reduced (*p* < 0.05) compared with those in the NC group, indicating that DSS induction led to a marked decrease in gut microbial richness. After BSP60 intervention, the ACE and Chao1 indices slightly increased in the L-BSP60 group, but these changes were not statistically significant relative to the MC group. The ACE and Chao1 indices in the H-BSP60 group were significantly higher than those in the MC group (*p* < 0.05), and the Chao1 index recovered to a level comparable with that in the NC group, suggesting that H-BSP60 effectively restores gut microbial richness in UC mice. Shannon and Simpson indices were used to evaluate microbial diversity. Compared with that in the NC group, the Shannon index was significantly decreased (*p* < 0.05) whereas the Simpson index was significantly increased (*p* < 0.05) in the MC group, indicating a pronounced reduction in gut microbiota diversity after DSS induction. After BSP60 intervention, the Shannon index in the L-BSP60 group was significantly higher than that in the MC group (*p* < 0.05), while the Simpson index displayed a decreasing trend. In the H-BSP60 group, the Shannon index was significantly higher than that in the MC group (*p* < 0.05) and showed no significant difference with that in the NC group, while the Simpson index was significantly reduced to near-normal levels (*p* < 0.05). These beneficial effects of BSP60 on gut microbial richness and diversity are consistent with the findings of Zou et al. [30], who reported that FMPS significantly increased the Chao1 and Shannon indices and decreased the Simpson index of the gut microbiota in mice with DSS-induced chronic colitis, thereby restoring gut microbial homeostasis. Thus, BSP60 dose-dependently mitigates the DSS-induced reduction in gut microbial richness and diversity, with the high dose exerting a potent effect on restoring gut microecological balance in UC mice.

Figure 6E shows a Venn diagram of amplicon sequence variants (ASVs). The five groups shared 74 common ASVs. The NC, MC, L-BSP60, and H-BSP60 groups contained 161, 127, 145, and 155 ASVs, respectively. BSP60 intervention increased the number of ASVs compared with that in the MC group, indicating that BSP60 enhances gut microbial species richness. Principal coordinate analysis (PCoA) was performed to assess differences in microbial composition among the five groups (Figure 6F). The L-BSP60 and H-BSP60 groups showed distinct clustering, indicating good intragroup reproducibility and stable microbial structure. Meanwhile, the PC group exhibited many dispersed sample points, suggesting great variability in microbial composition. Li et al. [33] reported that *Lycium barbarum* polysaccharide alleviated DSS-induced chronic UC by modulating the gut microbiota. Their PCoA analysis similarly showed convergence of the treatment group microbiota toward that of the control group, and their Venn diagram revealed many shared ASVs between the treatment and control groups.

Figure 6G shows that at the phylum level, the gut microbiota structure in the MC group was significantly disrupted compared with that in the NC group. In the NC group, Bacillota (formerly Firmicutes) accounted for 53.63%, Bacteroidota for 22.57%, and Verrucomicrobiota for 15.42%, making them the three dominant phyla. In the MC group, the abundance of Bacillota decreased to 46.47%, that of Verrucomicrobiota dropped sharply to 5.85%, that of Actinomycetota fell from 4.43% to 1.74%, and that of Pseudomonadota (formerly Proteobacteria) increased markedly from 3.75% to 14.98%. Pseudomonadota overgrowth can exacerbate intestinal inflammatory responses [34]. Chen et al.’s [35] study on *Anoectochilus roxburghii* polysaccharide revealed that Bacteroidota, Bacillota, Pseudomonadota, and Verrucomicrobiota together constituted over 90% of the microbial community in the control group. After BSP60 intervention, the abovementioned dysbiosis was markedly improved. In the H-BSP60 group, the abundance of Bacillota recovered to 54.75% (surpassing NC group level), that of Verrucomicrobiota rebounded to 8.10%, that of Actinomycetota decreased to 3.21%, and that of Bacteroidota decreased to 26.25%. The abundance of Pseudomonadota significantly decreased to 7.35%. The L-BSP60 group showed a similar trend, but the improvement was less pronounced than that in the H-BSP60 group. The promoting effect of BSP60 on the restoration of the Firmicutes phylum was also observed for the *Polygonatum kingianum* polysaccharide studied by Fan et al. [36]. In addition, Wu et al. showed that morel stem polysaccharides restored imbalance by increasing the relative abundance of Bacillota while reducing the relative abundances of Bacteroidota and Pseudomonadota. This finding is similar to our results.

**Figure 7:**
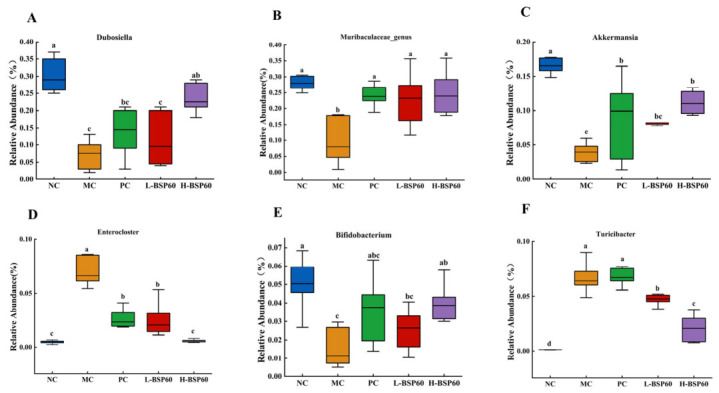
Effects of BSP60 on gut microbiota at the genus level in DSS-induced UC mice. (**A**): Relative abundance of Dubosiella; (**B**): Relative abundance of Muribaculaceae; (**C**): Relative abundance of Akkermansia; (**D**): Relative abundance of Enterocloster; (**E**): Relative abundance of Bifidobacterium; (**F**): Relative abundance of Turicibacter. The same letters indicate no significant difference (*p* > 0.05) and different letters indicate significant difference (*p* < 0.05).

Figure 6H and Figure 7 show the modulation of gut microbiota at the genus level by BSP60 intervention in UC mice. In the NC group, *Muribaculaceae* (21.40%), *Dubosiella* (24.23%), Akkermansia (14.55%), and Bifidobacterium (3.95%) were the dominant genera, all of which are beneficial bacteria [37]. Xiao et al. [38] reported that levan, a high-molecular-weight fructan, significantly enriched *Dubosiella*, and oral supplementation with *Dubosiella* effectively alleviated DSS-induced colitis. Feng et al. [39] demonstrated that *Muribaculaceae* is an SCFA-producing bacterial family, and its enrichment is correlated with improved intestinal barrier function and reduced allergic responses. *Muribaculaceae* can produce SFFAs and recover intestinal barrier function and the immune response, making it a promising “next-generation probiotic” [40]. In the MC group, the abundances of these beneficial genera decreased significantly (*Muribaculaceae* to 13.36%, *Dubosiella* to 8.10%, *Akkermansia* to 5.51%, and *Bifidobacterium* to 1.58%), whereas the abundances of *Ligilactobacillus* (20.14%), *Turicibacter* (4.00%), *Romboutsia* (5.31%), *Enterococcus* (4.59%), and *Bacteroides* (3.92%) significantly increased (*p* < 0.05). *Ligilactobacillus* species can affect the intestinal barrier function and increase intestinal immunity through various mechanisms [41], and its sharp increase in the MC group likely reflects a compensatory response to inflammation. The elevated abundances of *Turicibacter*, *Romboutsia*, and *Enterococcus* [42] are closely associated with intestinal inflammation and dysbiosis.

After BSP60 intervention, the abundances of *Muribaculaceae* (20.86%) and *Dubosiella* (19.93%) in the H-BSP60 group recovered to near-NC levels, while the abundance of Akkermansia (7.32%) was also significantly higher than that in the MC group. Meanwhile, the abundances of *Ligilactobacillus* (15.70%), *Turicibacter* (3.02%), *Romboutsia* (3.01%), *Enterococcus* (2.01%), and *Bacteroides* (0.17%) were partially normalized, with that of *Bacteroides* falling below the NC level. Furthermore, the abundances of *Enterocloster* and *Turicibacter* were significantly reduced after BSP60 intervention, indicating that BSP60 effectively inhibits the overgrowth of certain inflammation-associated bacterial genera. In addition, the abundance of Bifidobacterium was significantly reduced in the MC group but recovered in the BSP60 intervention groups, suggesting that BSP60 may have prebiotic-like effects that improve the growth and colonization of beneficial bacteria.

**Figure 8:**
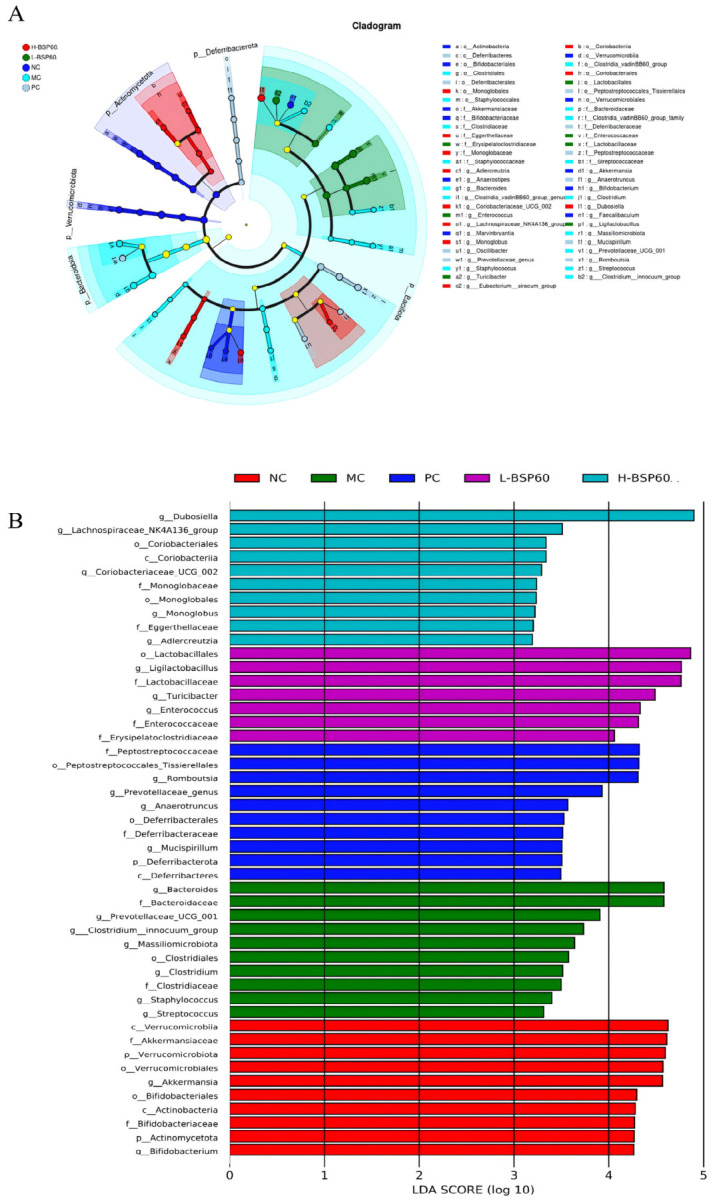
Effects of BSP60 on gut microbiota in DSS-induced UC mice. (**A**): Phylogenetic tree; (**B**): Bar chart showing the distribution of LDA values.

LEfSe can be used to identify biomarkers with statistically significant differences between groups, thereby revealing group-specific dominant taxa [43]. As shown in Figure 8A,B, the MC group was characterized by opportunistic pathogens as biomarkers, including *g_Bacteroides*, *f_Bacteroidaceae*, and *g_Clostridium_innocuum_group*. After BSP60 intervention, the L-BSP60 group showed significant enrichment of beneficial taxa such as *o_Lactobacillales*, while the H-BSP60 group was further enriched with functional bacteria, including the butyric acid-producing *g_Lachnospiraceae_NK4A136_group* and *g_Dubosiella*. Li et al. [44] demonstrated that the *Lachnospiraceae_NK4A136_group* possesses anti-inflammatory properties. The enrichment of beneficial bacteria such as *Lachnospiraceae_NK4A136_group* and *Dubosiella* by BSP60 is accordance to the findings of Yao et al. [45], who performed LEfSe analysis and demonstrated that citrus pulp polysaccharides selectively enriched *Lachnospiraceae_NK4A136_group* and *Dubosiella* in DSS-induced colitis mice. These results indicate that BSP60 dose-dependently shifts gut microbial biomarkers from a pathogenic to a beneficial phenotype and, at high doses, establishes a comprehensive and synergistic probiotic network.

### 3.6. Effects of BSP60 on Fecal SCFA in DSS-Induced Colitis Mice

BSP60 intervention markedly modulated fecal SCFA concentrations in DSS-induced colitis mice. Propionic acid, acetic acid, and butyric acid are the major SCFAs in the gut and play important roles in maintaining colonic epithelial integrity, modulating immune responses, and suppressing inflammatory responses [46].

**Figure 9:**
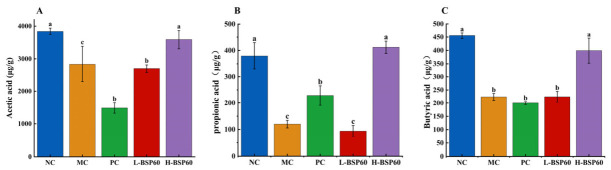
Effects of BSP60 on the SCFAs in DSS-induced UC mice. (**A**): Acetic acid content; (**B**): Propionic acid content; (**C**): Butyric acid content. The same letters indicate no significant difference (*p* > 0.05) and different letters indicate significant difference (*p* < 0.05).

Analysis of acetic acid revealed that the intestinal acetic acid content in the MC group was significantly reduced from 3840.91 μg/g to 1672.17 μg/g (*p* < 0.05) compared with that in the NC group, indicating that DSS induction markedly impairs the metabolic function of the gut microbiota (Figure 9A). The acetic acid levels in the PC and L-BSP60 groups were 2836.16 and 2953.76 μg/g, respectively; both were significantly elevated compared with that in the MC group (*p* < 0.05). The acetic acid content in the H-BSP60 group recovered to 3908.58 μg/g, attaining levels comparable with that in the NC group, indicating that high-dose BSP60 restores intestinal acetic acid production in UC mice. The propionic acid content in the MC group was significantly decreased from 379.39 μg/g in the NC group to 119.96 μg/g (*p* < 0.05). In the H-BSP60 group, the propionic acid content recovered to 412.17 μg/g, slightly exceeding NC level and suggesting strong restorative capacity (Figure 9B). Butyric acid is the primary energy source for intestinal epithelial cells and stimulates goblet cells. Its deficiency can result in impaired energy metabolism in intestinal epithelial cells and reduce the thickness of the mucus layer. In this study, the butyric acid content in the MC group was significantly decreased from 455.63 μg/g in the NC group to 200.15 μg/g (*p* < 0.05). The effect of BSP60 was dose dependent (Figure 9C). The butyric acid levels in the PC (223.56 μg/g) and L-BSP60 (223.90 μg/g) groups exhibited a slight recovery compared with that in the MC group. The butyric acid content in the H-BSP60 group recovered to 399.24 μg/g with no significant difference relative to the NC group, showing that high-dose BSP60 effectively rescues intestinal butyric acid production in UC mice. The restorative effects of BSP60 on SCFA profiles are consistent with previous studies, including those focusing on *Astragalus membranaceus* polysaccharides [47], *Phellinus linteus* acidic polysaccharides [31], and *Hirsutella sinensis* amylopectin-like polysaccharides [48].

Although this study provides robust evidence for the protective effects of BSP60 in the murine DSS-induced colitis model, caution must be exercised when extrapolating these results to humans. However, significant interspecies differences in drug-metabolizing enzymes and pharmacokinetic profiles, such that equivalent mg/kg doses do not ensure comparable systemic drug exposure; and the DSS model reflects acute chemical injury, whereas human UC is a chronic, relapsing disease with marked heterogeneity. Therefore, the appropriate human dose must be determined through rigorous safety and pharmacokinetic studies in both animals and humans.

## 4. Conclusions

A Bolete mushroom polysaccharide fraction (BSP60) was obtained. The anti-colitis effects of BSP60 and its underlying molecular mechanisms were evaluated using a DSS-induced mouse model of UC. BSP60 markedly ameliorated the basic symptoms, mitigated colonic histopathological injury, and exerted intestinal protective effects on UC mice by suppressing inflammatory responses and oxidative stress and restoring intestinal barrier function. Gut microbiota analysis revealed that BSP60 effectively ameliorated microbial dysbiosis in UC mice and enriched SCFA-producing bacteria. Metabolite profiling further confirmed its dose-dependent restoration of acetic acid, propionic acid, and butyric acid levels. This study elucidated the comprehensive mechanism by which BSP60 exerts anti-UC effects, providing a scientific basis for its application as a functional food ingredient to improve intestinal health.

## Figures and Tables

**Table 1:** DAI scoring sheet in mice.

Faecal Consistency	Weight Loss (%)	Faecal Bleeding	Rating
Granular stools	0	No bleeding	0
Semi-formed, paste-like stools	1–5%	A small amount of blood is visible on the surface of the stool	1
Loose stools	5–10%	There is visible blood in the stools (though the stools are largely well-formed)	2
Watery stools	10–20%	Blood in the stools	3
Liquid stools	≥20%	Significant bleeding	4

## Data Availability

Data will be made available on request.
